# Isolation and Characterisation of Phage‐Displayed scFv Antibodies Targeting PfHSP70 and PfLDH of *Plasmodium falciparum*

**DOI:** 10.1155/bri/2190193

**Published:** 2026-07-24

**Authors:** Nothando Lovedale Gasa, Lindiwe Khumbuzile Zuma, Thamsanqa Emanuel Chiliza, Lusisizwe Kwezi, Sifiso Duncan Luthuli, Ofentse Jacob Pooe

**Affiliations:** ^1^ Discipline of Biological Sciences, School of Agriculture and Science, University of KwaZulu-Natal, Durban, South Africa, ukzn.ac.za; ^2^ Chemicals Cluster, Council for Scientific and Industrial Research (CSIR), Pretoria, South Africa, csir.co.za; ^3^ Department of Biochemistry and Microbiology, Faculty of Science, Engineering and Agriculture, University of Venda, Private Bag X5050, Thohoyandou 0950, South Africa, univen.ac.za

**Keywords:** PfHSP70, PfLDH, phage display, *Plasmodium falciparum*, single-chain variable fragment (scFv)

## Abstract

*Plasmodium falciparum* is the most virulent human malaria parasite and is responsible for numerous deaths annually. The increasing resistance of *P. falciparum* to antimalarial drugs necessitates the development of improved diagnostic tools for timely malaria detection. Malaria biomarkers such as PfHSP70 and PfLDH are highly valuable for malaria detection because they are essential for parasite survival and are consistently expressed during infection. PfHSP70 is associated with the parasite’s stress adaptation and proteostasis mechanisms under febrile and drug‐induced conditions, while PfLDH plays a central role in glycolytic metabolism and redox balance. Their functional importance, parasite specificity and elevated expression during active infection make these proteins reliable molecular indicators for the sensitive and specific detection of *P. falciparum*, thereby supporting their potential application in rapid diagnostic and biosensing platforms for malaria surveillance and disease management. In this study, an M13 phage‐displayed single‐chain variable fragment (scFv) antibody library was used to screen for antibodies against recombinant PfHSP70 and PfLDH. Four rounds of biopanning were conducted to enrich high‐affinity binders, followed by ELISA, UV‐visible spectroscopy and microscale thermophoresis (MST) to evaluate specificity and binding affinity. Functional interactions were assessed in *Escherichia coli* expressing PfHSP70 or PfLDH. Recombinant PfHSP70 and PfLDH were successfully expressed and purified from *E. coli*, with approximately 50% of selected colonies demonstrating significant binding to both targets, confirming the enrichment of antigen‐specific phages. Specific phages were validated using ELISA and transmission electron microscopy (TEM). Selected scFvs exhibited strong target binding, with MST‐determined dissociation constants (Kd) of 7.47 μM for PfHSP70 and 3.64 μM for PfLDH. Exposure to scFv‐displaying phages impaired the survival of PfHSP70‐ and PfLDH‐expressing *E. coli* and induced spectral shifts consistent with protein–antibody interactions. This study validates phage display as a robust platform for isolating high‐affinity scFvs against *P. falciparum* targets. These binders have the potential to develop into low‐cost diagnostic tools, addressing the urgent need for novel diagnostic interventions. Nevertheless, further studies are required to confirm binding specificity and assess translational applicability.

## 1. Introduction

Despite substantial global efforts to eliminate and control malaria in recent years, it remains a major public health challenge. Recently, the World Health Organization reported 282 million malaria cases globally in 2025 with 610,000 malaria‐related deaths in 2024, compared to 598,000 deaths in 2023 [1]. These statistics underscore the persistent global burden of malaria and the urgent need for improved disease control strategies. Accurate malaria diagnosis remains a major challenge, particularly in remote and resource‐limited regions within malaria‐endemic areas. Current diagnostic approaches predominantly rely on microscopy and rapid diagnostic tests (RDTs). Although RDTs offer advantages such as rapid turnaround times, ease of use and reduced dependence on highly skilled personnel compared with microscopy, several limitations persist, including variable sensitivity, variability in parasite antigens and reduced detection at low parasitaemia levels [2]. These challenges highlight the critical need to identify novel diagnostic biomarkers and develop improved diagnostic strategies to enhance the sensitivity, specificity and overall reliability of RDT‐based malaria.

Identifying biomarkers for malaria is important in potentially improving diagnosis and, therefore, overall clinical outcomes. The lactate dehydrogenase enzyme of *Plasmodium falciparum* (PfLDH) has been widely utilised as an important antigen in the development of malaria RDTs [3]. Its application is largely attributed to its species‐specific detection of *Plasmodium*, high analytical sensitivity and compatibility with minimally invasive sample collection methods. Despite these advantages, several limitations are associated with the use of PfLDH as a diagnostic target. These include the potential for cross‐reactivity with other pathogens, variability in enzyme expression among different parasite strains and reduced detection sensitivity at low parasitaemia levels, which may result in false‐negative outcomes [4, 5]. Such limitations highlight the need for complementary diagnostic approaches to improve the accuracy, sensitivity and reliability of malaria detection [6]. Among the emerging targets, the heat shock Protein 70 of *P. falciparum* (PfHSP70) has attracted considerable interest due to its critical role in parasite survival and stress adaptation [7]. PfHSP70 functions as a central component of the parasite’s proteostasis network by facilitating ATP‐dependent protein folding, preventing the aggregation of misfolded proteins and stabilising stress‐destabilised client proteins during cellular stress conditions such as febrile episodes and antimalarial drug exposure [7–9]. These functional characteristics position PfHSP70 as a promising complementary biomarker for improving malaria diagnostic strategies.

Innovations such as multiplex assays and molecular techniques hold promise for enhancing sensitivity and specificity, ultimately facilitating the effective management and control of malaria. However, high costs, reliance on specialised laboratory infrastructure and lengthy turnaround times generally make them unsuitable for immediate, point‐of‐care clinical management in resource‐limited or highly endemic areas [10]. Phage therapy has made significant advances in recent years. Antigen–antibody binding affinities for identifying epitopes and mimotopes were among the earliest applications of phage display technology [11]. The discovery that large molecules could be displayed on phage facilitated the development of innovative molecular detection strategies, enabling detailed investigation of protein–protein interactions, structural properties and protein folding stability [12, 13].

The filamentous M13 bacteriophage is widely used in phage display studies. It carries a single‐stranded DNA genome encapsulated by thousands of copies of its major coat protein. Functional single‐chain variable fragment (scFv) antibody genes are typically fused to genes encoding minor coat proteins, enabling display on the phage surface [14–16]. Specific phage–antibody binders can be enriched through rounds of interactive affinity selection, known as biopanning [17]. The application of M13 phage display to isolate high‐affinity scFvs in this study expands the toolkit for malaria diagnostics. Building on these applications, the present study aims to assess the viability of the M13 phage display library for generating and selecting highly specific bacteriophages and scFvs targeting PfHSP70 and PfLDH, respectively. The goal is to explore their potential as diagnostic tools and research reagents. Further optimisation and integration with existing technologies could pave the way for the development of rapid, cost‐effective and robust detection assays suitable for resource‐limited settings where the malaria burden remains significant.

## 2. Materials and Methods

The selected malarial proteins used in this study were PfHSP70 (accession number: PF3D7_0818900) and PfLDH (accession number: PF3D7_1324900), with molecular weights of 70 and 34.9 kDa, respectively. The *M13* phage display library, *E. coli* TG1 cells and M13KO7 (2 × 10^12^ CFU/mL) helper phages.

### 2.1. Recombinant Expression and Purification of PfHSP70

In this study, the pQE30/PfHsp70‐1 plasmid construct encoding PfHsp70 and the pKK223‐3/PfLDH plasmid encoding PfLDH were used to overexpress both recombinant proteins in *Escherichia coli* BL21 (DE3) cells, as previously reported [8, 18]. The cells containing the recombinant genes were grown in 1000 mL of LB broth at 37°C in the presence of 50 μg/mL kanamycin. At A600 = 0.6, the expression was induced using 1 mM isopropyl‐β‐D‐1‐thiogalactopyranoside (IPTG). Twenty‐four hours post‐IPTG induction, the cells were harvested by centrifugation, and the pellet was suspended in LEW buffer (50 mM NaH2PO4, 300 mM NaCl–HCl, pH 7.4). The crude cell extract was further lysed by sonication for 2 min (6 cycles of 20 s ON/OFF at 60% amplitude and 50% duty cycle, on ice, with a 3‐mm probe). The cellular debris was then removed by centrifugation at 8000 × g for 20 min at 4°C, and the crude lysate extract was used for PfHSP70 purification. The supernatant was loaded onto a nickel‐charged Protino Nitrilotriacetic Acid (Ni‐NTA; Macherey‐Nagel GmbH, Germany) column for 1 h on ice to enable efficient affinity binding of the His‐tagged protein. Following the manufacturer’s protocols, the Ni‐NTA was then washed using LEW. The bound protein was eluted using an LEW buffer containing 250 mM imidazole. The eluted protein fractions were then visualised by 12% sodium dodecyl sulphate–polyacrylamide gel electrophoresis (SDS‐PAGE). Recombinant protein production was confirmed using α‐His horse‐radish peroxidase (HRP)–conjugated antibodies (Sigma‐Aldrich, USA) at a 1:2000 dilution; colourimetric western blot assays were conducted using a PVDF membrane (Merck, Germany) and visualised using the 3,3′,5,5′‐tetramethylbenzidine (TMB) liquid substrate system for membranes (Sigma‐Aldrich, USA).

### 2.2. Preparation of TG1 Mid‐Logs and Selection of ScFvs Against PfHSP70 From the M13 Library by Panning

The growth medium was inoculated with TG1 from an in‐house glycerol stock prepared and grown overnight at 37°C at 220 rpm. The overnight culture was diluted 1:100 into fresh 2 × TY medium and grown at 37°C until the OD at 600 nm reached 0.5 (mid‐log phase). Panning was performed as described by Van Wyngaardt et al. [12], with minor modifications. A 96‐well plate was coated with 20 μg/mL PfHSP70 overnight at 4°C [12, 19]. The coated wells were washed three times with PBS and three times with PBST. The nonspecific sites in the wells were blocked with 2% MPBS at room temperature (RT) for 1 h. Simultaneously, the M13 phage stock was preincubated in 4% MPBS for an hour. The preincubated library was added to the washed wells and incubated at RT for 90 min. The wells were washed 20 times with PBST and then 20 times with PBS. Bound phages were eluted by adding 150 μL 100 mM triethylamine (TEA) and neutralised with 75 μL 1M Tris‐HCl (pH 7.4), and then added to 5 mL TG1 mid‐log cells. The mixture was incubated at 37°C for 30 min at rest, followed by an additional 30 min with shaking at 100 rpm. An aliquot was used to infect the cells for phage titration, while the remainder was centrifuged for 10 min at 4000 rpm. The supernatant was removed, and the pellet was resuspended in 600 μL 2 × TY medium. The cells were plated on TYE agar plates containing 100 μg/mL ampicillin and 2% glucose medium (TY/AG) and incubated overnight at 30°C.

### 2.3. Phage Rescue Investigations

Glycerol stocks were prepared by scraping bacteria from plates in TY/AG, then mixing the cells with 60% glycerol (3:1 ratio). For phage amplification, the glycerol stock was used to inoculate fresh TY/AG medium to an initial OD of 0.03–0.05. The remaining glycerol stock was stored at −80°C. The inoculated medium was grown until the optical density at 600 nm (OD600 nm) reached 0.5. From this exponentially growing culture, a portion was infected with M13KO7 helper phage at a 20:1 ratio and incubated at 37°C for 30 min at rest, followed by another 30 min of shaking at 100 rpm. Bacterial cells were pelleted by centrifugation at 4000 rpm for 10 min and then resuspended in 2 × TY supplemented with 100 μg/mL ampicillin and 50 μg/mL kanamycin. The culture was grown overnight at 30°C with shaking at 240 rpm.

### 2.4. Phage Precipitation and scFvs Specificity Analysis Using ELISA

Cells were centrifuged at 10,000 rpm for 10 min, and the phage in the supernatant was precipitated by adding 1/5 volume of 20% (v/v) PEG/NaCl and incubating for 1 h on ice. Phages were collected by centrifugation at 10,000 rpm for 15 min. The pellet was resuspended in PBS after centrifugation. These phages were then used for the second round of panning. Four successive rounds of panning were performed. To verify whether the anti‐PfHSP70 ScFv recognises recombinant PfHSP70, an enzyme‐linked immunosorbent assay was performed. A 150‐μL aliquot of protein was used to coat a Polysorp ELISA well, which was then incubated overnight at 4°C. The coated wells were washed three times with PBST, then three times with PBS, and finally blocked with MPBS for 1 h at RT. The washing step was repeated, followed by 90 min incubation with anti‐PfHSP70 ScFv supernatants. The washing step was repeated, followed by another 1 h incubation with an anti‐mouse mAb conjugated to HRP (Roche) diluted 1:1000 in MP/PBS/0.05%T. A TMB substrate (50 μL) was added to each well. The signal was measured spectrophotometrically at 450 nm after the reaction was stopped with 2 M H_2_SO_4_.

### 2.5. Q‐Dot Conjugation of Phage‐Displayed scFvs for Detection of Antigen Proteins

Quantum dots (QDs) were conjugated to phage‐displayed antibodies as described by the manufacturer (Thermo Fisher Scientific, US). Qdot 655 ITKTM amino (PEG) QDs are used in conjugation with PfHSP70/PfLDH‐specific phage‐displayed scFvs. The commercial Qdot 655 ITKTM amino (PEG) QDs are CdSe/CdS QDs with an amine‐derivatised polyethene glycol (PEG) outer coating that reacts directly with amine‐reactive groups or carboxylic acids on proteins, making them an ideal choice for binding to our test phages [20]. The bound Qdots‐conjugated phages were then investigated for their potential to selectively bind target antigens using fluorescence spectroscopy.

## 3. Results

### 3.1. Successful Recombinant PfHSP70 and PfLDH Expression and Purification

**FIGURE 1:**
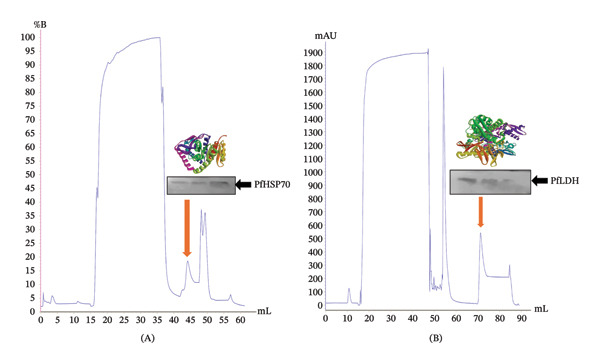
Purification and confirmation of recombinant malaria proteins. His‐tagged 70 kDa PfHSP70 (A) and 34.9 kDa PfLDH (B) were purified by His‐Trap affinity chromatography using an AKTAstart system. Purified proteins were confirmed by western blot using anti‐His HRP‐conjugated antibody.

Recombinant PfHSP70 and PfLDH were successfully expressed in *E. coli* BL21 (DE3) cells. The expression was carried out in 1 L of LB medium supplemented with ampicillin (100 mg/L) for PfHSP70 or kanamycin (50 mg/L) for PfLDH at 37°C with shaking at 180 rpm for 24 h. Protein expression was induced with 1 M IPTG once the cultures reached an OD_600_ of 0.4. After harvesting the cells by centrifugation, they were lysed in LEW buffer and sonicated. The supernatant was then purified using an AKTAstart system with 1 mL His‐Trap columns. The presence of purified proteins was confirmed by western blotting with an HRP‐conjugated anti‐His monoclonal antibody, demonstrating the successful purification of PfHSP70 and PfLDH (Figure 1).

### 3.2. Selection of scFv Antibodies From the M13 Phage Display Library

The M13 phage display library underwent four rounds of biopanning against PfHSP70 and PfLDH, as previously described. During each round, unbound phages were removed, and bound phages were eluted, amplified in *E. coli* TG1 cells and used in the next selection round. Enrichment was assessed by ELISA using a mouse anti‐M13 monoclonal antibody, followed by HRP‐conjugated anti‐mouse IgG. The antigen concentration immobilised on plates was halved in successive rounds to increase selection stringency.

**FIGURE 2:**
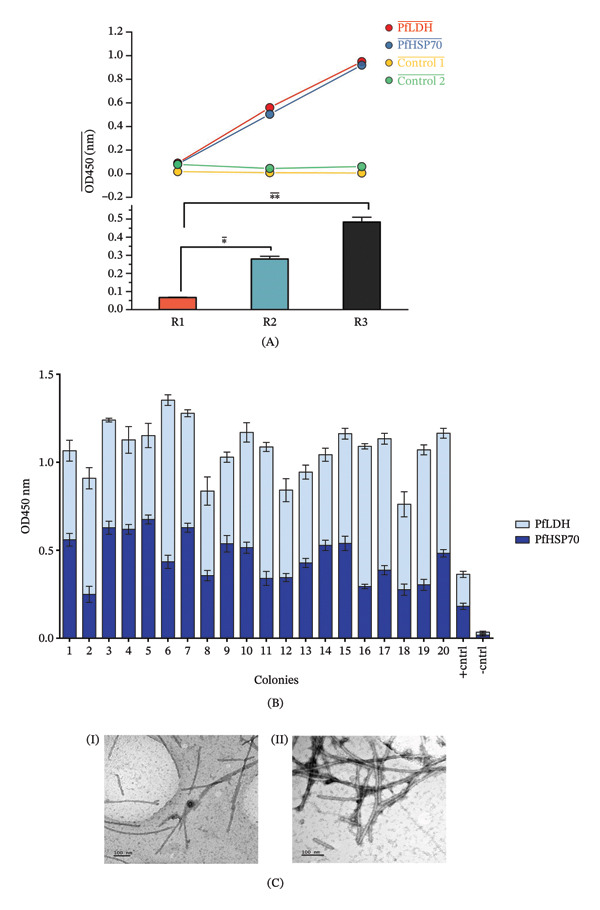
Selection and enrichment of specific scFv antibodies by biopanning. (A) Enrichment of precipitated phage‐displayed scFvs over three rounds of panning. The upper panel shows absorbance values, while the lower panel presents enrichment as mean ± SE. Asterisks indicate significant differences (*p* < 0.05); (^∗∗^) indicates a significant difference relative to (^∗^). (B) ELISA analysis of selected colonies binding to target proteins. (C) TEM images showing the filamentous morphology for PfLDH (I) and PfHSP70 (II).

ELISA screening of 20 individual clones revealed that 11 (55%) and 9 (45%) showed strong reactivity to PfHSP70 and PfLDH, respectively (Figure 2A). *E. coli* TG1 and the original M13 library stock were used as negative controls. Colonies with absorbance values > 0.5 were classified as high binders and selected for further analysis. The absorbance threshold above 0.5 indicates a significant level of binding activity, ensuring that only the strongest interacting clones are chosen for subsequent studies. The selection process improves the reliability of the findings by concentrating on clones that exhibit the most promising reactivity to the target proteins. The selected high binders were subsequently subjected to additional assays to evaluate their specificity and affinity.

A paired comparison, multiple panel graph was constructed. The upper layer contains a 3‐point line series plot showing the absorbance obtained against the PfHSP70 and PfLDH target proteins after 3 rounds of biopanning. The lower layer contains mean +  SE bars of enrichment for the biopanning assay; Friedman ANOVA was used to investigate statistical analysis, with *p* < 0.05 considered significant. Progressive enrichment of PfHSP70‐ and PfLDH‐specific binders were demonstrated with each round (Figure 2C), supporting the utility of the M13 phage library for isolating high‐affinity scFvs. Transmission electron microscopy confirmed the presence of filamentous phage displaying scFvs specific to PfHSP70 and PfLDH (Figure 2C).

### 3.3. Direct Interaction Between Phage‐Displayed scFv Antibodies and Their Respective Targets

#### 3.3.1. UV‐Visible Spectroscopy

**FIGURE 3:**
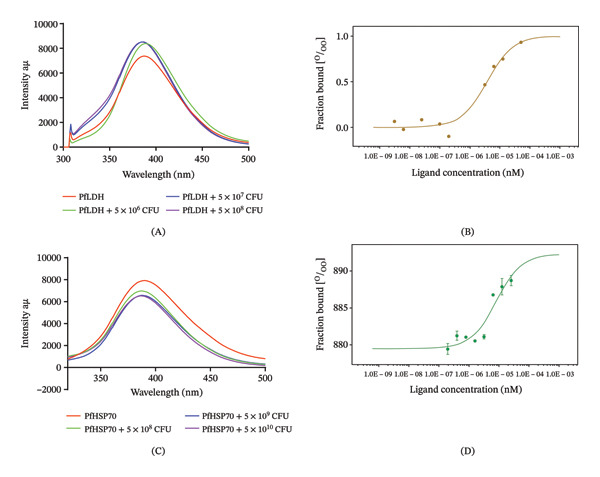
Direct interaction of phage‐displayed scFv and their target proteins. (A, C) UV‐Vis spectroscopic analysis of PfLDH and PfHSP70 tertiary structure in the presence of varying phage concentrations. (B, D) MST binding curves for PfLDH–anti‐PfLDH and PfHSP70–anti‐PfHSP70 interactions, yielding dissociation constants Kd 3.64 ± 1.28 μM and Kd 7.47 ± 6.18 μM, respectively.

UV‐visible spectroscopy further confirmed binding interactions. The PfHSP70 spectrum showed a concentration‐dependent hypsochromic effect, accompanied by a hypsochromic (blue) shift in the emission maximum. The progressive decrease in fluorescence intensity suggested quenching of intrinsic fluorophores, likely arising from antibody‐antigen complex formation and reduced solvent accessibility of aromatic residues upon binding [21]. Concurrently, the blue shift indicates a transition of fluorophores into a more hydrophobic microenvironment, consistent with conformational rearrangements induced by specific interaction between PfHSP70 and anti‐PfHSP70 phage‐displayed scFv [22]. Collectively, these spectral changes provide strong evidence for stable, specific binding, supporting the formation of a PfHSP70‐scFv complex and confirming the functional recognition capability of the selected scFv clone (Figure 3C). The hypsochromic shift observed in the UV‐visible spectroscopy indicates a successful interaction between the scFvs and the target protein. In contrast, the PfLDH spectrum exhibited a concentration‐dependent hyperchromic effect with a blue shift (Figure 3A). The increase in fluorescence intensity suggests enhanced excitation or reduced quenching of intrinsic fluorophores, indicative of exposure of aromatic residues upon interaction with anti‐PfLDH phage‐displayed scFv [23]. The concomitant blue shift implies that these fluorophores experience a more hydrophobic local environment, consistent with binding‐induced structural rearrangements that stabilise the antigen–antibody complex. Together, the hyperchromic response and hypsochromic shift support a distinct binding mechanism compared to PfHSP70, potentially reflecting differences in epitope accessibility, binding orientation or PfLDH’s conformational flexibility upon scFv engagement. This hyperchromic shift in PfLDH suggests that scFv binding may alter its structural integrity, potentially compromising its function. Such changes highlight the distinct mechanisms by which scFvs can interact with different target proteins, emphasising their specificity.

#### 3.3.2. Microscale Thermophoresis (MST) Analysis

MST was employed to determine the binding affinities of pooled phage–antigen interactions. MST detects changes in protein mobility upon binding, influenced by alterations in size, charge, and hydration shell. The dissociation constant (Kd) for the PfHSP70–anti‐PfHSP70 interaction was 7.47 ± 6.18 μM (Figure 3B), while PfLDH–anti‐PfLDH binding yielded a Kd of 3.64 ± 1.28 μM (Figure 3D). The dissociation constants (Kd) reflect the strength of binding interactions between PfHSP70 and its antibody, as well as between PfLDH and its antibody. A lower Kd value indicates a stronger interaction, with PfLDH binding to its antibody more robust than PfHSP70 [24]. The comparative analysis of the binding interactions between PfHSP70 and anti‐PfHSP70, as well as between PfLDH and anti‐PfLDH, reveals significant insights into their respective affinities. The Kd value of 7.47 ± 6.18 μM for the PfHSP70–anti‐PfHSP70 interaction indicates a relatively weaker binding affinity when compared to the PfLDH–anti‐PfLDH interaction, which has a Kd value of 3.64 ± 1.28 μM. These results underline the importance of Kd values in assessing the strength of biochemical interactions; a lower Kd reflects a stronger binding affinity. This points out the importance of PfLDH as a more effective target for therapeutic strategies against malaria. This synthesis not only emphasises the distinct binding properties of these proteins but also highlights their implications for drug development to combat parasitic diseases. These micromolar affinities may also indicate strong interactions between the scFv‐displaying phages and their respective targets.

### 3.4. Specificity Evaluation and Application of Phage‐Displayed scFvs With Target Antigens

#### 3.4.1. Inhibition of Target Protein‐Expressing *E. coli* by Respective scFv

**FIGURE 4:**
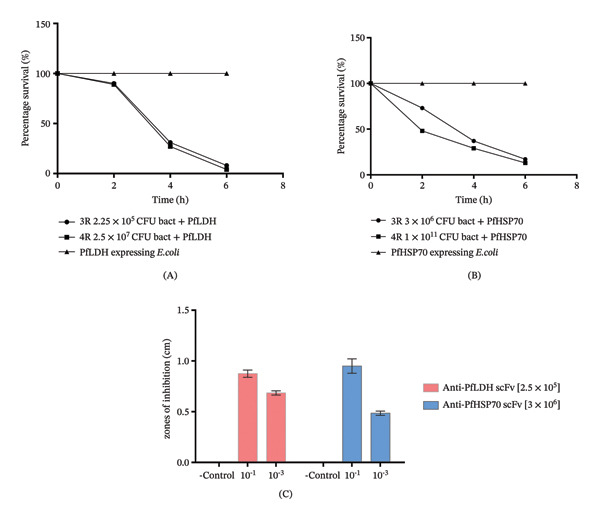
Interaction of phage‐displayed scFv antibodies with their target antigens. (A, B) Time‐dependent survival of PfLDH‐ and PfHSP70‐expressing *E. coli* following exposure to scFv‐displaying phages enriched in the third and fourth panning round. (C) Disk diffusion assay demonstrating growth inhibition of PfLDH‐ and PfHSP70‐expressing cells; the experiment was conducted in duplicates.

The functional effect of pooled scFv‐displaying phages (from Rounds three and four) was evaluated by incubating *E. coli* BL21 (DE3) cells expressing PfHSP70 or PfLDH with varying phage concentrations at an OD_600_ of 0.4. Growth was monitored for 6 h postinduction. The results demonstrated a significant decrease in *E. coli* growth upon exposure to higher concentrations of phage‐displayed scFvs. This suggests a strong interaction between the scFvs and their corresponding target antigens. In both instances, cell survival decreased over time in the presence of scFv‐displaying phages, indicating antigen–antibody interaction and potential functional interference (Figure 4A, B). These results suggest that the phage‐displayed scFvs may serve as promising leads for future therapeutic or inhibitory strategies against malaria [25].

To further evaluate the functionality of the selected phage‐displayed scFv antibodies, a disk diffusion assay was performed using *E. coli* BL21 cells expressing either PfLDH or PfHSP70. Cells were spread onto LB agar plates supplemented with kanamycin, after which discs containing anti‐PfLDH or anti‐PfHSP70 phage‐displayed scFvs were placed on the agar surface, with PBS serving as a negative control (Figure 4C). Distinct zones of growth inhibition were observed around discs containing the corresponding phage‐displayed scFvs, whereas no inhibition was detected around the PBS control. Furthermore, the size of the inhibition zones increased with increasing scFv concentration, indicating a concentration‐dependent effect.

These findings demonstrate that the selected phage‐displayed scFv antibodies retain their functionality and specifically interact with their respective target antigens expressed in *E. coli*. The absence of inhibition in the PBS control supports the specificity of these interactions. The observed target‐specific and concentration‐dependent effects suggest that the selected scFvs possess favourable binding characteristics, highlighting their potential utility as recognition elements for malaria diagnostic applications. Specific antigen recognition is particularly important for diagnostic development, as it can enhance assay sensitivity while minimising cross‐reactivity with nontarget molecules [26–28].

#### 3.4.2. Qdot‐Conjugated Phage Application

**FIGURE 5:**
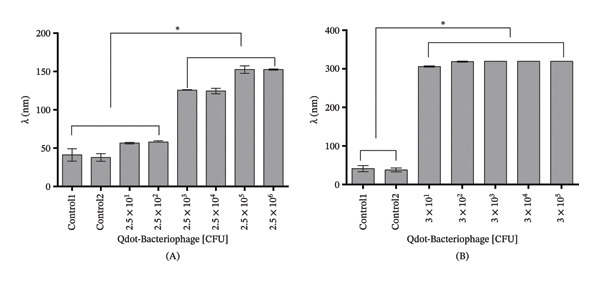
Detection of target proteins using quantum dot–conjugated phage‐displayed scFv antibodies. (A, B) Detection of PfLDH (A) and PfHS70 (B) using quantum dot–conjugated phage‐displayed scFv antibodies specific to each target. Asterisks indicate statistically significant differences (*p*  <  0.05).

Fluorescence intensity was measured at 627 nm using a high‐speed custom setting. This wavelength corresponds to the red emission channel commonly used for quantifying binding events, protein expression or concentration‐dependent fluorescence responses (Figure 5). Fluorescence spectral analysis demonstrated successful detection of PfLDH and PfHSP70 by their respective Qdot‐labelled phage‐displayed scFv antibodies. For Qdot‐anti‐PfLDH/PfLDH interaction (Figure 5A), a progressive rise in fluorescence response with increasing bacteriophage concentration was observed, resulting to a clear dose‐dependent elevation in emission wavelength.

The graph shows that the assay had not yet reached saturation, suggesting a requirement for higher phage density to achieve maximal target interaction. In contrast, the Qdot‐anti‐PfHSP70/PfHSP70 interaction (Figure 5B) produced a markedly higher fluorescence shift, with a strong response evident even at the lowest concentration tested. Beyond this point, additional increases in phage‐displayed scFv produced minimal further change, reflecting early saturation consistent with higher effective binding affinity for PfHSP70. Overall, these results confirm that both labelled scFv antibodies successfully recognise their respective targets, with anti‐PfHSP70 scFv exhibiting a stronger interaction.

## 4. Discussion and Conclusion

This study demonstrates the successful isolation, characterisation and functional validation of M13 phage‐displayed scFv antibodies targeting two biologically and clinically important *P. falciparum* proteins, PfHSP70 and PfLDH. Through iterative biopanning, ELISA screening, spectroscopic analysis, MST and functional inhibition assays, we show that phage display is a robust and versatile platform for generating antigen‐specific binders against essential malaria targets.

Spectroscopic analyses provided mechanistic insight into the nature of scFv‐antigen interactions that are relevant to detection performance. The hypsochromic shift and fluorescence quenching observed for PfHSP70 indicate that antibody binding induces a more compact and hydrophobic microenvironment, consistent with conformational stabilisation. From a diagnostic perspective, this suggests that PfHSP70 presents structurally constrained epitopes upon binding, which may enhance specificity but could limit signal intensity or accessibility in assay formats [29]. In contrast, the spectral behaviour of PfLDH, marked by a hyperchromic effect alongside a hypsochromic shift, supports a different interaction mode characterised by increased fluorophore exposure or reorientation. This implies a more accessible and flexible binding interface, which is advantageous for diagnostic applications as it can facilitate stronger signal generation and improved detection sensitivity. These distinct binding behaviours highlight the importance of target‐dependent assay design. The comparatively strong affinity and enhanced fluorescence response of PfLDH support its suitability as a robust diagnostic marker, particularly for platforms requiring high sensitivity, such as rapid tests or fluorescence‐based assays. Meanwhile, PfHSP70 may offer a complementary value in applications where binding specificity is prioritised. These spectroscopic findings are strongly supported by MST data, which revealed micromolar binding affinities for both targets, with PfLDH‐scFv interactions exhibiting a lower dissociation constant than PfHSP70‐scFv interactions. Importantly, both interactions fall within a biologically relevant affinity range for diagnostic and inhibitory applications [30].

Functional relevance was further demonstrated through the ability of scFv‐displaying phages to specifically recognise and bind *E. coli* expressing PfHSP70 or PfLDH. The concentration‐dependent responses observed in liquid culture and disk diffusion assay confirm that target recognition is both specific and quantifiable, supporting its application in diagnostic platforms. These findings collectively indicate that the selected scFvs are not only high‐affinity binders but also reliable biorecognition elements suitable for sensitive antigen detection. The scFv‐mediated targeting of PfHSP70 and PfLDH highlights their potential as diagnostic biomarkers, enabling selective detection of malaria‐associated proteins. This specificity supports the development of targeted detection systems capable of distinguishing infected from noninfected samples with high accuracy [31]. Furthermore, the integration of scFv‐displaying phages with QDs enabled sensitive fluorescence‐based detection of both antigens. The Q‐dot–anti‐PfHSP70 system demonstrated rapid signal saturation at low phage concentrations, indicating strong binding efficiency and high detection sensitivity. Overall, these results support the use of scFv‐displaying phage constructs as effective tools for developing next‐generation diagnostic assays for malaria, offering rapid, sensitive and specific detection of key parasitic antigens.

While the findings of this study are encouraging, several avenues remain to be explored to advance the translational relevance of these scFv binders. These include epitope‐mapping studies to precisely define the binding sites of the selected scFv on PfHSP70 and PfLDH. Techniques such as peptide arrays, alanine‐scanning mutagenesis or structural modelling combined with docking simulations were used to clarify whether the observed hyperchromic and hypsochromic effects arise from binding near catalytic, regulatory or structurally flexible regions. This information would be invaluable for rational optimisation of binding affinity and specificity. Affinity maturation of the scFvs through additional rounds of mutagenesis and selection could also be employed to improve binding strength, particularly for PfHSP70, where MST revealed a comparatively higher Kd. Future works should also conduct specificity studies against homologous human proteins, particularly HSP70 and LDH isoforms for assessing potential cross‐reactivity. Furthermore, in vitro studies should be extended to *P. falciparum* parasite cultures to confirm scFv binding and functional effects in a biologically relevant system. Assessing parasite growth inhibition, protein localisation or modulation of stress responses would provide direct evidence of antiplasmodial potential beyond heterologous expression *in E. coli*. Nonetheless, the preliminary Qdot‐conjugated phage approach used in this study warrants further optimisation for diagnostic development studies and for integration into lateral flow assays, microfluidic platforms or multiplex detection systems.

## Funding

The authors acknowledge the National Research Foundation of South Africa (Grant No. 145396) and the South African Medical Research Council (SAMRC), through its Division of RCD‐EIP, for funding awarded to O.J.P.

## Conflicts of Interest

The authors declare no conflicts of interest.

## Data Availability

The data that support the findings of this study are available on request from the corresponding author. The data are not publicly available due to privacy or ethical restrictions.
